# IMPLEMENTATION OF THE GOALS OF CARE DECISION AID IN NURSING HOMES: AN EVIDENCE-BASED INTERVENTION

**DOI:** 10.1093/geroni/igz038.022

**Published:** 2019-11-08

**Authors:** Latarsha Chisholm, Laura Hanson, Sheryl Zimmerman, Cherie Rosemond, Bryan Weiner, Eleanor S McConnell

**Affiliations:** 1 University of Central Florida, Orlando, Florida, United States; 2 University of North Carolina, Chapel Hill, North Carolina, United States; 3 Cecil G. Sheps Center for Health Services Research, Chapel Hill, North Carolina, United States; 4 University of Washington, Seattle, Washington, United States; 5 Duke University, Durham, North Carolina, United States

## Abstract

Nursing homes (NH) must implement best practices to improve care. The purpose of this study is to understand NH characteristics that help or hinder implementation of the Goals of Care (GOC) intervention, an evidence-based decision aid to guide decision-making in advanced dementia. Study design was a cross-sectional staff survey at 11 NHs in North Carolina that participated in the GOC trial. Questions measured the dependent variable of implementation effectiveness (IE) (the consistency and quality of use of the GOC intervention). NH organizational characteristics were measured using publicly available data and administrator surveys. Averages were obtained, IE and NH factors above the average were ranked as high and vice versa. Analysis consisted of pattern matching logic, predicted results are compared to actual results, using within-case and cross-case analyses. NHs with high IE were expected to have the majority (five or more) of NH characteristics to be high to confirm the within (within-case analysis) and between (cross-case analysis) relationships. Within-case analysis expected results were met in 5 NHs, with 3 highs (IE high and five or more characteristics were high) and 2 lows (IE low and five or more characteristics were low). Among three NHs with high IE, the following characteristics were high: Medicare and SNF/ICF beds. Across 6 of the 11 NHs, high total beds and percent of White residents were related to high IE. NHs with enhanced resources may have fewer challenges implementing innovations compared to resource constrained NHs. Implementation strategies should account for resource limitations to promote successful implementation.

